# WT1 Peptide Cancer Vaccine for Patients with Hematopoietic Malignancies and Solid Cancers

**DOI:** 10.1100/tsw.2007.119

**Published:** 2007-05-29

**Authors:** Yoshihiro Oka, Akihiro Tsuboi, Olga A. Elisseeva, Hiroko Nakajima, Fumihiro Fujiki, Manabu Kawakami, Toshiaki Shirakata, Sumiyuki Nishida, Naoki Hosen, Yusuke Oji, Ichiro Kawase, Haruo Sugiyama

**Affiliations:** ^1^ Department of Respiratory Medicine, Allergy and Rheumatic Diseases, Osaka University Graduate School of Medicine, Japan; ^2^ Department of Cancer Immunotherapy, Osaka University Graduate School of Medicine, Japan; ^3^ Department of Functional Diagnostic Science, Osaka University Graduate School of Medicine, Japan

**Keywords:** WT1, cancer, immunotherapy, vaccine, clinical trial

## Abstract

Wild-type Wilms' tumor gene *WT1* is expressed at a high level in hematopoietic malignancies including acute leukemia, chronic myelogenous leukemia, and myelodysplastic syndromes, as well as in various kinds of solid cancers. Human cytotoxic T lymphocytes (CTLs), which could specifically lyse *WT1*-expressing tumor cells with HLA class I restriction, were generated *in vitro*. It was also demonstrated that mice immunized with the WT1 peptide rejected challenges by *WT1*-expressing cancer cells and survived with no signs of autoaggression to normal organs that physiologically expressed *WT1*. Furthermore, we and others detected IgM and IgG WT1 antibodies in patients with hematopoietic malignancies, indicating that the WT1 protein was highly immunogenic, and that immunoglobulin class-switch-inducing, WT1-specific, cellular immune responses were elicited in these patients. CD8^+^ WT1-specific CTLs were also detected in peripheral blood or tumor-draining lymph nodes of cancer patients. These results provided us with the rationale for elicitation of CTL responses targeting the WT1 product for cancer immunotherapy. On the basis of these findings, we performed a phase I clinical trial of a WT1 peptide cancer vaccine for the patients with malignant neoplasms. These results strongly suggested that the WT1 peptide cancer vaccine had efficacy in the clinical setting because clinical responses, including reduction of leukemic blast cells or regression of tumor masses, were observed after the WT1 vaccination in patients with hematopoietic malignancies or solid cancers. The power of a tumor-associated-antigen (TAA)-derived cancer vaccine may be enhanced in combination with stronger adjuvants, helper peptide, molecular-target-based drugs, or some chemotherapy drugs, such as gemcitabine, which has been revealed to suppress regulartory T-cell function. In contrast, reduction of WT1 peptide dose may be needed for the treatment of patients with hematological stem cell diseases, because rapid and strong destruction of malignant cell-sustained hematopoiesis before recovery of normal hematopoiesis may lead to pancytopenia in these patients.

## INTRODUCTION

Despite recent progress in surgical, chemotherapeutic, and radiotherapeutic approaches, cancer is still one of the diseases that is difficult to treat and cure, especially in patients with advanced stages of the disease. Therefore, new therapeutic strategies are required. One of the recent developments in this cancer treatment field is immunotherapy that targets tumor-associated antigens (TAAs).

*WT1,* Wilms' tumor gene, was isolated as a gene responsible for tumorigenesis of a childhood renal neoplasm, Wilms' tumor[1,2]. This gene encodes a zinc finger transcription factor involved in the regulation of cell proliferation and differentiation, in apoptosis, and in organ development[3,4,5,6,7]. The *WT1* gene was categorized at first as a tumor-suppressor gene on the basis of the findings, such as intragenic deletions or mutations in Wilms' tumor, germline mutations in patients with leukemic predisposition syndromes, and *WT1*-mediated growth suppression of Wilms' tumor cells. However, we recently proposed that the wild-type *WT1* gene performs an oncogenic rather than a tumor-suppressor function in leukemogenesis and tumorigenesis in various types of solid cancers, based on the following accumulated evidence: (1) high expression levels of the wild-type *WT1* gene in various kinds of human malignant neoplasms including hematopoietic malignancies, such as leukemia and myelodysplastic syndromes (MDS), and solid cancers[8,910,11,12,13,14,15,16,17,18,19,20,21,22,23,24,25] (Table 1) (as far as we have determined, there are no qualitative differences such as point mutations in *WT1* genes between tumor cells and normal cells); (2) growth inhibition of human leukemic or solid cancer cells by the treatment with *WT1* antisense oligonucleotides[17,26]; and (3) growth promotion and differentiation block of murine myeloid progenitor cells by constitutive expression of wild-type *WT1* gene[27,28].

**TABLE 1**
**WT1-Expressing Malignancies**

| |
|---|
| Hematopoietic malignant disorders |
| Acute myeloid leukemia (AML) |
| Acute lymphocytic leukemia (ALL) |
| Chronic myelogenous leukemia (CML) |
| Myelodysplastic syndromes (MDS) |
| Non-Hodgkin lymphoma (NHL)[a] |
| Multiple myeloma (MM)[b] |
| Solid cancers |
| Lung cancer |
| Breast cancer |
| Head and neck squamous cell carcinoma |
| Bone and soft-tissue sarcoma |
| Thyroid cancer |
| Esophageal cancer |
| Colorectal adenocarcinoma |
| Pancreatic ductal adenocarcinoma |
| Primary astrocytic tumors |

[a] Expression of WT1 in NHL cells was very recently demonstrated. See text for details.

[b] Although WT1 expression level in MM is not so high, MM cells are very susceptible to WT1-specific CTLs. See text for details.

$CD8^+$ cytotoxic T lymphocytes (CTLs) are the most important effector cells for antitumor immune responses, and they recognize TAA-derived peptides that are "processed" and presented on the tumor cell surface in association with major histocompatibility complex (MHC) class I molecules, leading to the killing of the tumor cells[29,30]. Clinical evidence for the effectiveness of antitumor immune responses has been obtained in several clinical settings, including graft-vs.-leukemia (GVL) effect after allogeneic hematopoietic stem cell transplantation[31].

Taken together, these findings strongly suggested that the WT1 protein might be a promising target antigen for cancer immunotherapy against hematopoietic malignancies as well as various kinds of solid cancers[32,33]. Tumor escape from immune surveillance as a result of down-regulation of *WT1* expression is unlikely to occur[32,33] because expression of *WT1* at a high level seems to have an essential role in leukemogenesis or tumorigenesis, and seems to be required to maintain the transformed phenotype[17,26,27,28]. This is a theoretical advantage for using an oncogenic function-bearing protein like the *WT1* product as a target antigen for cancer immunotherapy.

In this article, we will review recent investigations, including ours and those of other groups, aiming at the development of cancer immunotherapy targeting the Wilms' tumor gene *WT1* product. Furthermore, since we have performed a phase I clinical study of a WT1 peptide cancer vaccine, and observed clinical responses in some cases after the vaccination, we will also summarize the result with discussion about other groups' clinical study result, and briefly describe the clinical courses of some of the clinical response-positive patients. Finally, we will describe a perspective for cancer immunotherapy, particularly peptide cancer vaccine.

## *IN VITRO* GENERATION AND CHARACTERIZATION OF HUMAN WT1-SPECIFIC CTLs

### Identification of HLA Class I–Restricted CTL Epitopes of WT1

Several groups, including ours, succeeded in the generation of human WT1-specific CTLs (Table 2). We identified Db126 (RMFPNAPYL, also called WT126: underlined letters represent anchor-motifs for HLA-A*0201) and WH187 (SLGEQQYSV) as epitopes that could induce WT1-specific CTLs with the restriction of HLA-A*0201, which was the most frequent HLA class I type in Caucasians and also frequent in Japanese[32,34,35]. This result was confirmed by another group that used a unique "allorestricted CTL approach" to generate the WT1-specific CTLs[36]. This approach seems to be very useful to circumvent immunologic tolerance to TAAs that are mostly self-proteins[36]. WT235 (CMTWNQMNL, originally called WT1-T2; an underlined letter represents an anchor-motif for HLA-A*2402) was identified as an epitope that could induce WT1-specific CTLs with the restriction of HLA-A*2402, which was the most frequent HLA class I type in Japanese and also frequent in Caucasians[37,38]. It was recently demonstrated that the WT235 peptide could induce not only HLA-A*2402, but also HLA-A*0201–restricted, WT1-specific CTLs[39,40]. A new HLA-A*2402–restricted CTL epitope, RWPSCQKKF (F at position 9 is an anchor-motif for HLA-A*2402), was also identified recently[41].

The CTLs that were generated from peripheral blood T cells by stimulation with the WT1 peptides killed endogenously *WT1*-expressing tumor cells including leukemia and solid cancer cells[32,34,35, 36,37,38,39,40,41,42,43], indicating that the epitope peptides were "processed" from the WT1 protein in the tumor cells, followed by the presentation on the cell surface in association with HLA class I molecules to be recognized by the CTLs.

All the CTL epitopes listed in Table 2 had a common structure in that they had at least one anchor residue for the peptide-binding HLA class I molecules, as described in the first part in this chapter. It would be very useful if we could predict the strength of the antitumor effect of each peptide as a cancer vaccine. However, it is impossible at this moment to predict the strength according to the sequences. Furthermore, it is difficult to compare the immunogenic ability of the peptides identified as CTL epitopes

**TABLE 2**
**CTL Epitopes that can Elicit WT1-Specific CTLs**

| |
|---|
| HLA-A*0201 restriction |
| RMFPNAPYL |
| SLGEQQYSV |
| CMTWNQMNL[a] |
| HLA-A*2402 restriction |
| CMTWNQMNL[a] |
| CYTWNQMNL[b] |
| RWPSCQKKF |

[a] This peptide was revealed to elicit WT1-specific CTLs with restriction of both HLA-A*0201 and HLA-A*2402.

[b] This peptide is not derived from natural WT1 protein, but was modified from peptide-a. See text for details.

Both HLA-A*0201 and HLA-A*2402 are frequent HLA class I types in human population.

(Table 2) because the CTL-induction system for the identification of CTL epitopes, such as stimulator cells, target cells, and peripheral blood mononuclear cell (PBMC) donors who have individual T-cell repertoires, is different depending on the report. For example, one group demonstrated that Db126-induced CTLs showed stronger cytotoxic activity against *WT1*-expressing leukemia cells than WH187-induced CTLs, while another group demonstrated that WH187-induced CTLs showed stronger cytotoxic activity against *WT1*-expressing colon cancer cells than Db126-induced CTLs[32,43]. One of the reasons that makes identification of CTL epitopes and prediction of immunologic activity of the candidate peptides difficult may be that the binding affinity of the peptides to MHC molecules does not necessarily correlate with the immunologic ability of the peptides because the high binding affinity of peptides to MHC molecules can induce tolerance *in vivo*, and the peptide/MHC complex-reactive T cells are prone to be eliminated or inactivated[34,44,45], and the remaining T-cell repertoires that can recognize the peptide/ MHC complex vary among individual patients.

## Enhancement of Induction and Killing Activity of Human WT1-Specific CTLs using a Modified WT1 Peptide

We introduced a single amino-acid substitution, M→Y, into the first anchor residue at position 2 of the natural immunogenic HLA-A*2402–restricted WT1 peptide, WT235[46] (Table 2). This substitution markedly increased the binding affinity of the peptide to the HLA-A*2402 molecule[46]. As expected from the increased binding affinity of the peptide, the modified WT235 peptide (CYTWNQMNL) elicited WT1-specific CTLs more efficiently than the natural peptide, WT235[46]. It was considered that the modified peptide-pulsed antigen-presenting cells strongly activated CTL precursors that could recognize the modified peptide/HLA-A*2402 complex, and that the CTLs induced by the stimulation with the modified WT235 killed not only the modified WT235 peptide-pulsed target cells, but also natural WT235 peptide-pulsed target cells, and, more importantly, endogenously *WT1*-expressing tumor cells with HLA-A*2402 restriction and at higher efficiency than natural WT235-induced CTLs[46]. Thus, this modified WT1 peptide was expected to be very useful for a cancer vaccine targeting WT1 for HLA-A*2402–positive patients.

### No Inhibition of Normal Hematopoietic Cell Colony Formation by Human WT1-Specific CTLs

Since several kinds of normal cells, including hematopoietic progenitor cells, physiologically express *WT1*, it is critical to know whether WT1 peptide-specific CTLs cause damage to normal cells physiologically expressing *WT1* if we intend to apply the WT1-specific CTL-based immunotherapy in the clinical setting[33,34,35]. It was demonstrated that HLA-A*0201– or HLA-A*2402–restricted, WT1-specific CTLs, which killed *WT1*-expressing tumor cells, did not inhibit colony formation by normal bone marrow cells[36,37,47], indicating that the CTLs did not attack *WT1*-expressing normal hematopoietic progenitor cells[34,35,36,37]. The selective CTL killing of leukemic blasts, but not normal hematopoietic progenitor cells, both of which express *WT1*, may be explained by the difference in the *WT1* expression level between malignant and normal hematopoietic cells[36]. Other possible explanations may be differences in the mechanism of processing of WT1 protein and/or surface presentation of WT1 peptide, or differences in the susceptibility of the cell membranes to CTL-producing molecules, such as perforin, between malignant and normal cells. Further studies are needed to address this issue.

### Evidence of *In Vivo* Tumoricidal Activity of Human WT1-Specific CTLs using Immunodeficient Mouse Model

In order to see whether human WT1-specific CTLs have tumoricidal activity *in vivo*, the WT1-specific CTLs and the restricting HLA allele-matched *WT1*-expressing leukemia cells[47,48] or solid cancer cells[49] were transplanted in immunodeficient mice. In these experiments, inhibition of the cancer cell growth due to attack by the CTLs was observed[47,48,49]. It was also shown that the CTLs did not inhibit engraftment of normal $CD34^+$ hematopoietic stem cells[48]. These results strongly suggested that human WT1-specific CTLs had the ability to attack cancer cells, but not normal cells, in *in vivo* as well as *in vitro* settings.

## SPONTANEOUS IMMUNE RESPONSES AGAINST WT1 PROTEIN IN CANCER PATIENTS

Several investigations, including ours, demonstrated that immune responses against the WT1 protein were naturally elicited in patients with hematopoietic malignancies, indicating that the WT1 protein had high immunogenicity in those patients[50,51,52,53,54,55,56]. These findings provided us with a rationale for developing cancer immunotherapy that targets WT1.

### Humoral Immune Responses against WT1 Protein in Patients with Hematopoetic Malignancies

We demonstrated that more than half of the patients with hematopoetic malignancies, such as acute myeloid leukemia (AML), chronic myelogenous leukemia (CML), and myelodysplastic syndromes (MDS), responded *in vivo* to leukemia cell–derived WT1 protein and produced both IgM- and IgG-type WT1 antibody[52], indicating that not only WT1-specific B cells, but also T cells that were needed to induce class switch of WT1 antibody from IgM to IgG, were activated in the patients. These results were basically compatible with those of other groups[50,51]. Intensive analysis of MDS patients revealed that class switch of WT1 antibody from IgM to IgG occurred along with the disease progression from refractory anemia (RA) to refractory anemia with excess of blast (RAEB), and further to RAEB in transformation (RAEB-t)[52], namely, with an increase in tumor burden. The stronger stimulation of the immune system by the higher tumor burden (more amount of WT1 protein) in patients with advanced stages of MDS might more strongly activate WT1-specific T cells to induce the class switch of the WT1 antibody. Furthermore, in AML patients, the WT1 antibody disappeared after the achievement of complete remission (CR), suggesting that the reduced stimulation of the immune system by the lesser amount of WT1 protein with decreased tumor burden in CR gave rise to discontinuation of WT1 antibody production[52]. These findings indicated that the WT1 protein was highly immunogenic and could elicit not only humoral, but also cellular, immune responses in the patients[50,51,52].

We also demonstrated more recently that Th1-type WT1 antibodies, such as IgG1, IgG2, and IgG3, were significantly increased in patients with leukemia and MDS compared to those in healthy volunteers, whereas Th2-type WT1 antibody, such as IgG4, did not increase in these patients[53]. These results showed that Th1-biased WT1-specific immune responses, which were essentially needed for cancer immunotherapy targeting WT1, might be elicited in patients with hematopoietic malignancies.

### CD8$^+$ T Cell Responses against WT1 Protein in Patients with Malignancies

The question of whether leukemia-related, antigen-specific, CD8$^+$ T-cell responses spontaneously occurred in AML or CML patients with HLA-A*0201 was investigated[54,55]. T cells recognizing HLA-A*0201/WT1 peptide complex could be detected at a relatively high rate by ELISPOT assay or intracellular IFN-γ detection assay in PBMCs of AML patients[54]. This result provided direct evidence for spontaneous cellular T-cell responses against WT1 protein in patients with hematopoietic malignancies, such as AML. It is interesting that the responses to leukemia-related antigens, including WT1, were higher in CML patients after hematopoietic stem cell transplantation (HSCT) than in those before HSCT[55]. The increased responses to TAAs such as WT1 in CML patients after HSCT may be one of the possible explanations for the GVL effect of allogeneic HSCT. Using quantitative reverse transcription-PCR to measure IFN-γmRNA production by CD8$^+$ T cells, Rezvani et al. also detected T-cell responses directed against HLA-A*0201–restricted WT1 epitopes in leukemia patients and healthy donors[56].

As for solid tumors, functional HLA-A*0201/WT1 tetramer-binding T cells could be expanded from tumor-draining lymph nodes, but not from peripheral blood, in patients with early-stage breast cancer[57]. This result strongly suggested that WT1 protein–responding CTLs were enriched or activated in the tumor-draining lymph nodes.

These data are results for HLA-A*0201–positive patients. We showed that HLA-A*2402/WT1 tetramer-binding CD8$^+$ T cells were detected in peripheral blood of HLA-A*2402–positive patients with leukemia or solid tumors at higher frequencies than that of healthy donors[58].

Taken together from accumulated experimental data described above, we could speculate a scenario of spontaneous elicitation of WT1-specific immune responses in humans, as summarized in Fig. 1.

## MOUSE *IN VIVO* MODELS FOR WT1 CANCER VACCINE

As mentioned above, several groups, including ours, succeeded in the generation of human WT1-specific CTLs from PBMCs *in vitro*. It is also very important to determine whether the WT1 protein could serve as a tumor rejection antigen *in vivo*. To address this issue, mouse models are essential because we can evaluate both the efficacy and the side effects of WT1 vaccination *in vivo* using these models.

### Tumor Rejection with No Autoaggression by WT1 Peptide or *WT1* cDNA Vaccination in Prophylactic Mouse Models

When we immunized C57BL/6 mice with activated, antigen-presenting cells (APCs) pulsed with Db126 peptide (a. a. 126-134, RMFPNAPYL: underlined letters represent anchor motifs for H-2D$^b$), which had

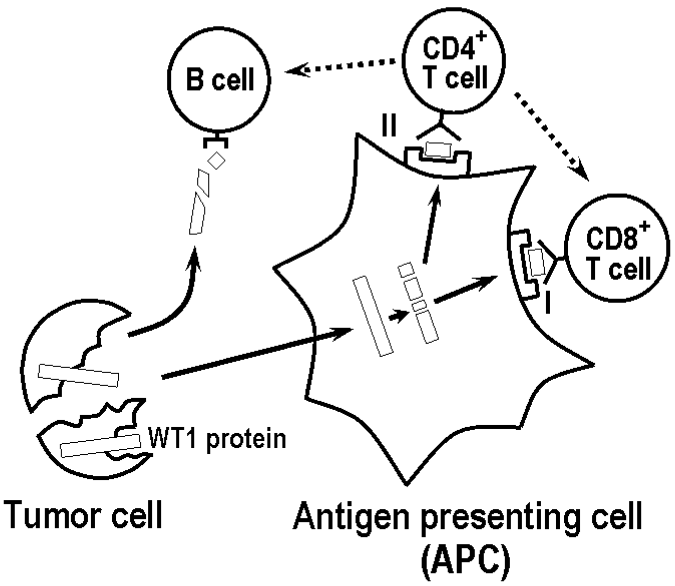

**FIGURE 1.** Spontaneous elicitation of immune responses against the WT1 protein. Tumor cell–derived WT1 protein stimulates B lymphocytes to produce anti-WT1 antibody, while the WT1 protein is ingested by APCs, such as dendritic cells (DCs), and is processed in them, followed by presentation of the WT1 protein–derived peptides in association with HLA molecules on the surface of the APCs. WT1 peptide/HLA class I complexes stimulate $CD8^+$ T cells to make WT1-specific CTLs. WT1 peptide/HLA class II complexes stimulate $CD4^+$ T cells to make WT1-specific, helper T cells, which more activate CTLs and B cells, respectively. The B cells "helped" by the helper T cells produce class-switched IgG-type, as well as IgM-type, anti-WT1 antibody.

relatively high binding affinity for $H\text{-}2D^b$ molecules and is by chance identical to the peptide used for the induction of human HLA-A*0201–restricted WT1-specific CTLs, the WT1 peptide–specific CTLs were shown to be generated from the spleen cells of the immunized mice[33]. The induced CTLs lysed *WT1*-expressing tumor cells as well as the WT1 peptide–pulsed target cells. Furthermore, the immunized mice rejected challenges of *WT1*-expressing tumor cells more efficiently than nonimmunized mice[33]. To evaluate the risk of autoaggression by the immunization against normal *WT1*-expressing cells, namely, the side effects of WT1 peptide vaccination, the WT1-expressing normal tissues, such as pleura and kidney of the immunized mice, were examined histologically. The tissues showed normal structure and cellularity in all of the mice examined and no pathological changes caused by the immune response, such as lymphocyte infiltration or tissue destruction and repair, were observed, indicating that the CTLs against WT1 did not give damage to normal cells that physiologically expressed *WT1*[33].

We also developed a *WT1* DNA vaccination system[59]. When plasmid DNA encoding mouse full-length WT1 protein was injected intramuscularly into C57BL/6 mice, the vaccinated mice elicited CTLs against the *WT1*-expressing tumor cells[59]. Furthermore, the vaccinated mice rejected the challenges of *WT1*-expressing tumor cells and about 50% of the vaccinated mice survived, while none of the mice injected with control plasmid DNA survived[59]. *WT1* DNA vaccination, as well as WT1 peptide vaccination, gave no pathological damage to physiologically *WT1*-expressing tissues, such as kidney[59]. In addition, no difference in the colony numbers of CFU-GEMM, CFU-GM, CFU-G, or BFU-E was observed between the immunized and nonimmunized mice, indicating that bone marrow hematopoetic progenitor cells that physiologically expressed *WT1* were not damaged by the CTLs induced by the *WT1* DNA vaccination[59]. These findings indicated that the *WT1* DNA-vaccinated mice could reject *WT1*-expressing tumor cells with no signs of autoaggression caused by the induced WT1-specific CTLs.

### Tumor Rejection with No Autoaggression by WT1 Peptide Vaccination in a Therapeutic Mouse Model

In order to examine whether WT1 peptide vaccination had the potency to reject tumor cells in therapeutic settings, we developed the following novel system. Intradermal injection of *Mycobacterium bovis* bacillus Calmette-Guerin cell wall skeleton (BCG-CWS) as an adjuvant into C57BL/6 mice, followed by intradermal injection of WT1 peptide (Db126 peptide) at the same site on the next day, generated WT1-specific CTLs and led to the rejection of *WT1*-expressing leukemia or lung cancer cells that had been implanted into the mice prior to the vaccinations[60]. The mice vaccinated with the WT1 peptide in combination with BCG-CWS survived significantly longer than the control mice, namely, mice with no treatment, mice vaccinated with the WT1 peptide alone, or mice injected with BCG-CWS alone[60]. No damage in physiologically *WT1*-expressing normal tissues, such as kidney and bone marrow, in the mice vaccinated with the peptide and BCG-CWS was observed. BCG-CWS was shown to be effective for eliciting TAA (WT1 in this case)-specific immunity to reject tumor challenges when it was injected in combination with TAA-derived peptides[60]. Because BCG-CWS is now being used in human settings[61,62,63], it may be used as a strong adjuvant in combination with WT1 peptide vaccine for cancer immunotherapy in the near future.

## CLINICAL TRIALS OF WT1 PEPTIDE CANCER VACCINE

At a "laboratory experiment" level, as described so far in this article, we and others obtained results that demonstrated that WT1 was one of the most suitable (strong and safe) tumor antigen candidates for cancer immunotherapy. On the basis of these results, we performed a phase I clinical trial of cancer immunotherapy targeting the WT1 protein, namely, a trial of the WT1 peptide cancer vaccine for HLA-A*2402–positive patients with leukemia, MDS, lung cancer, or breast cancer[58,64,65]. We used HLA-A*2402–restricted WT1 peptide with Montanide ISA51 as adjuvant because it is one of the most commonly and safely used adjuvants[66,67], and is therefore considered to be appropriate for phase I clinical trials in which we mainly evaluate the toxicity of the peptide vaccine. We injected 0.3, 1.0, or 3.0 mg of WT1 peptide intradermally per body at 2-week intervals. We observed clinical responses in some cases after the vaccination, as reported recently[58,64,65].

A patient with overt leukemia from MDS, who had HLA-A*2402, was injected intradermally with 0.3 mg of an HLA-A*2402–restricted, 9-mer WT1 peptide emulsified with Montanide ISA51 adjuvant[64] (Fig. 2). The vaccination resulted in an increase in WT1-specific CTLs in peripheral blood[64], followed by a rapid reduction in leukemic blast cells and WT1 levels in the bone marrow. Severe leukocytopenia was also observed. It was speculated that the leukocytopenia was induced because most normal-appearing peripheral blood cells in MDS patients were derived from *WT1*-expressing transformed hematopoietic stem/progenitor cells[64,68], which were attacked by the vaccination-induced CTLs. It is likely that leukocytopenia was specific to MDS patients in which hematopoiesis was mainly sustained by transformed hematopoietic cells. Therefore, the leukocytopenia was considered to be a clinical effect as well as an adverse effect[58,64]. Another MDS patient vaccinated with the WT1 peptide and Montanide ISA51 adjuvant also had a similar clinical course[64]. These two patients are the first reported cases in which WT1 peptide vaccination was performed and showed clinical responses of reducing leukemic blast cells, i.e., tumor burden. In some *de novo* AML patients who were in hematological complete remission, but had minimal residual disease at microscopic and/or molecular levels, a decrease in residual leukemic cells and/or *WT1* expression level which reflected minimal residual deseases was observed (Fig. 3). In contrast to MDS patients, leukocytopenia was not observed because normal hematopoiesis remained enough in the *de novo* AML patients[58].

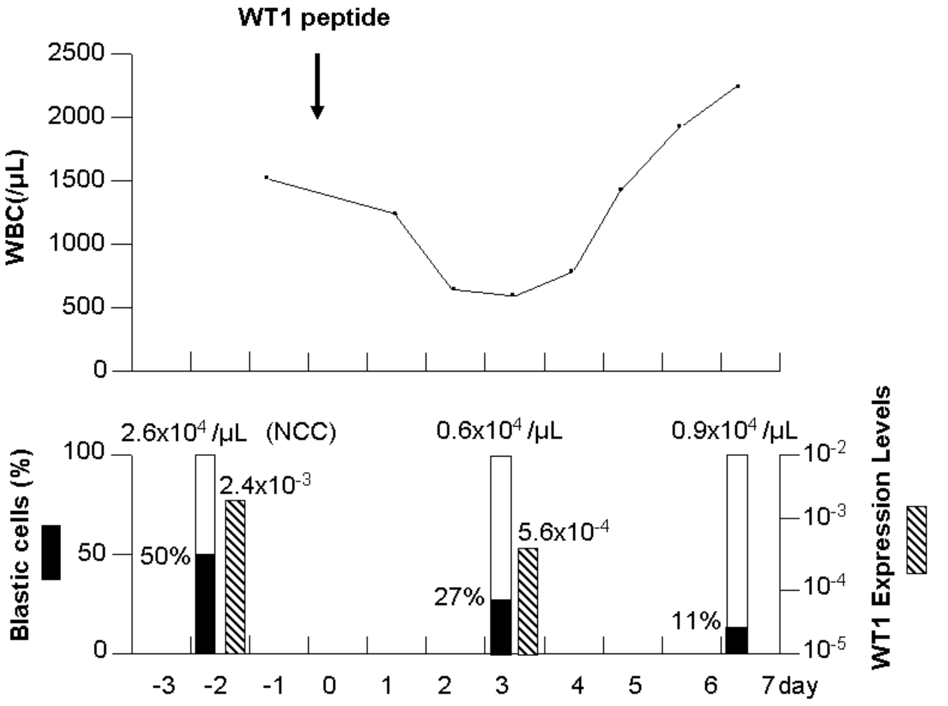

**FIGURE 2.** Reduction of blast cells and WT1 expression level in bone marrow, concomitant with leukocytopenia, in an MDS-derived AML patient treated with the WT1 peptide vaccination. Speculation for mechanism of the simultaneous occurrence of leukemic blast cell reduction and leukocytopenia is described in the text. WBC count was recovered by administration of G-CSF and steroid to stop the vaccination-mediated immune reaction. NCC: nucleated cell count. This figure was modified from that in Oka, Y. et al. (2003) *Int. J. Hematol*. **78,** 56–61.

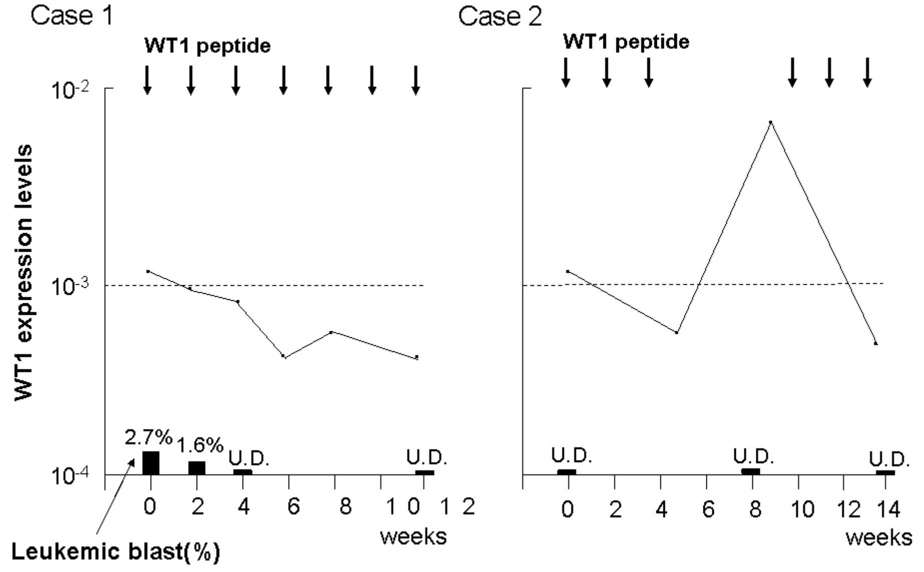

**FIGURE 3.** Reduction of leukemic blast cells and/or WT1 expression levels in bone marrow without leukocytopenia in *de novo* AML patients treated with WT1 peptide vaccination. In case 2, decrease of WT1 expression level was observed with the WT1 peptide vaccination, followed by increase of the WT1 level after cessation of the vaccination. The WT1 level decreased again after the restart of the vaccine. Correlation between the WT1 peptide vaccination and the WT1 level reduction was strongly suggested. U.D.: undetectable. This figure was modified from that in in Oka, Y. et al. (2004) *Proc. Natl. Acad. Sci. U.S.A.* **101,** 13885–13890.

In this phase I clinical trial, of the 14 patients with leukemia, including *de novo* leukemia and MDS, seven patients showed clinical responses, such as a reduction of leukemic cells and/or WT1 expression level[58].

Independently of our group, another group also reported an AML patient with HLA-A*0201 in whom complete remission was achieved and maintained by the vaccination with HLA-A*0201–restricted WT1 peptide (Db126)[32,69]. They vaccinated the patient with the WT1 peptide with GM-CSF and KLH[69]. No toxicity against the function of *WT1*-expressing normal tissues, including bone marrow and kidney, was observed[69].

In addition to leukemia patients, we observed clinical responses to the WT1 vaccination in solid cancer patients. A patient (HLA-A*2402) with lung cancer, that was completely resistant to chemotherapy and progressive, was injected with 0.3 mg of HLA-A*2402–restricted WT1 peptide[65]. Chorioembryonic antigen (CEA), a tumor marker, significantly decreased and the largest mass among the multiple tumors in the lung fields was slightly reduced after the vaccination[65]. Another patient (HLA-A*2402) with lung cancer, in whom operative resection of tumor masses in lung was performed, was injected with 0.3 mg of HLA-A*2402–restricted WT1 peptide after the reincrease of sialyl Lewis (x) (SLX), a tumor marker, which indicated regrowth of the tumor after the operation. SLX began to decrease after the first vaccination and returned to a normal level[65]. In this patient, WT1 vaccination has been repeated with maintained clinical benefit for more than 1 year without significant adverse effects, indicating that the induced WT1-specific CTLs did not give damage to physiologically *WT1*-expressing normal tissues, including normal hematopoietic stem/progenitor cells and kidney podocytes.

In two of the breast cancer patients enrolled in this study, metastatic tumors regressed after the WT1 vaccination[58]. One example is shown in Fig. 4, in which metastatic tumors in the lung regressed. In another patient, the WT1 vaccination has been repeated for more than 1.5 years because the clinical benefit persisted during the time. The patient could not eat food because of ileus caused by the metastatic tumors in the colon before the WT1 vaccination. After the vaccination, the tumor regressed[58], accompanied by improvement of appetite and relief from meteorism. The patient could spend her daily life without limitation of daily activities, including oral ingestion, during most of the vaccination period.

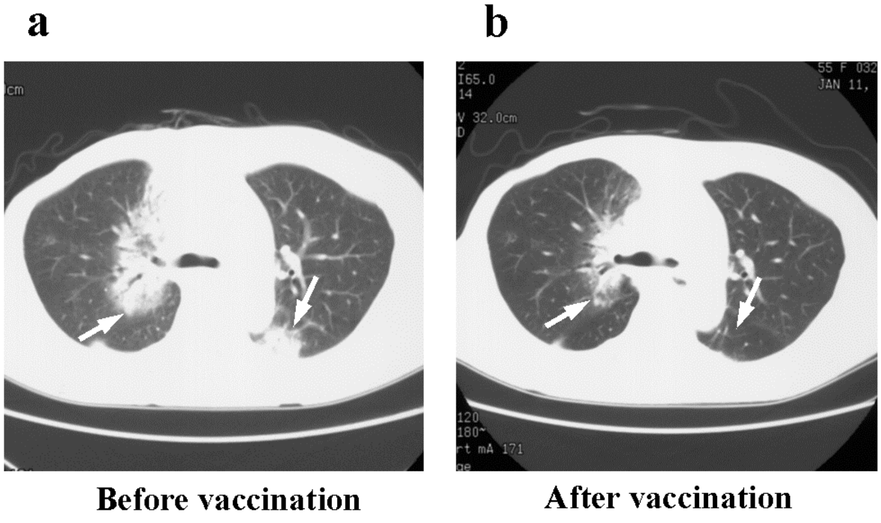

**FIGURE 4.** A representative case with solid cancer that showed clinical response to the WT1 peptide vaccination. This case is a breast cancer patient with lung metastasis. The metastatic tumors (indicated with arrows) detected before the vaccination (a) regressed after the vaccination (b). This figure was modified from that in Oka, Y. et al. (2004) *Proc. Natl. Acad. Sci. U.S.A.* **101,** 13885–13890. Copyright National Academy of Sciences, USA.

Taken together for solid cancer patients, of the 12 patients with lung or breast cancer in the phase I clinical trial, five patients showed clinical responses, such as decrease in tumor size or tumor marker[58]. Treatment of more patients with more various kinds of solid cancer is needed to evaluate precisely the clinical efficacy of the WT1 vaccination for solid cancer patients. How to evaluate clinical responses in immunotherapy, whose action mechanism is completely different from that of chemotherapy, is also an important issue. We would like to discuss this issue later in “Future Directions”.

In the phase I clinical study, a significant correlation between immunological (increase in WT1/HLA-tetramer-positive CTLs) and clinical response was observed in the 19 evaluable patients, indicating that WT1-specific CTLs induced by WT1 vaccination played an important role in the clinical response[58].

No damage to physiologically *WT1*-expressing normal tissues was observed in the patients who had clinical responses after the vaccination, as it was expected from preclinical data[33,36,37,47,59,60]. Therefore, it was considered that the induced CTLs attacked *WT1*-expressing tumor cells, but not physiologically *WT1*-expressing normal cells. Possible mechanisms to explain this observation were mentioned before in "No Inhibition of Normal Hematopoietic Cell Colony Formation by Human WT1-Specific CTLs". Furthermore, inflammation existing in tumor sites, but not in normal tissues, might contribute to the migration of the WT1-specific CTLs to tumor cells and to the activation of the CTLs, which could then become strong enough to kill tumor cells.

We are now conducting a phase I/II trial of weekly injection of WT1 peptide vaccine for patients with various kinds of solid cancers in addition to lung or breast cancers that were included in the former phase I clinical trial. The phase I part composed of the first 10 patients has been finished[70]. The vaccination-related systemic toxicities were not observed. Of the 10 patients, one patient (glioblastoma) exhibited a partial response (PR) and five additional patients had stable disease (SD) during the treatment period of 12 weeks.

## NEW TARGET DISEASES FOR WT1-DIRECTED CANCER IMMUNOTHERAPY

Recently, two kinds of hematological malignancies, non-Hodgkin lymphoma (NHL) and multiple myeloma, were presented as candidates for target diseases for WT1-directed immunotherapy.

Although WT1 expression in NHL cells has not been studied, Drakos et al. assessed for WT1 expression in 167 NHLs using immunohistochemical methods[71]. WT1 protein was frequently detected, primarily in the cytoplasm, of a subset of high-grade NHLs[71] (Table 1).

The expression levels of *WT1* mRNA in myeloma cells were significantly lower than those in acute leukemia cells, leading to a tentative conclusion that multiple myeloma was not a good target disease for WT1-directed cancer immunotherapy. However, a recent investigation showed that myeloma cells were lysed efficiently by WT1-specific CTLs in an HLA-restricted manner in spite of the low expression level of *WT1* mRNA in the cells[72]. The authors demonstrated that the high sensitivity of the myeloma cells to CTL-mediated cytotoxicity appeared to be because of the high susceptibility of the cell membranes to perforin.

Thus, WT1-directed cancer immunotherapy targeting NHL and multiple myeloma should also be planned (Table 1).

## FUTURE DIRECTIONS

Based on data accumulated from basic (preclinical) research, we and others have started clinical trials of WT1 peptide cancer vaccine as translational research. Fortunately, it was revealed that the WT1 vaccine had clinical efficacy at least for some patients with malignant neoplasms. Our goal is to establish a WT1 protein–targeted cancer vaccine that is strong enough to eradicate residual cancer cells and can be applied to patients with various kinds of malignant neoplasms by enhancing the power of the WT1 cancer vaccine. Strategies to enhance immunologically the power of TAA-derived peptide cancer vaccines may include (1) finding or developing more strong adjuvants, (2) using HLA class II–restricted helper epitopes that can enhance the induction and activation of CTLs and make "memory" CTLs in combination with HLA class I–restricted CTL epitopes[73,74,75,76], and (3) using multiple epitope peptides[77,78]. Efforts for the enhancement of cancer vaccine power using these strategies are under way.

Combined usage of cancer vaccines and other kinds of drugs, including molecular-target-based drugs such as imatinib[78], or chemotherapy drugs[79,80,81], might be another strategy to develop a novel modality for anticancer action. It was recently reported that addition of a multipeptide vaccine against BCR-ABL–derived p210 fusion protein to conventional treatment (imatinib) in patients with CML induced improved cytogenetic responses[78]. Blockade or removal of regulatory T cells by using approaches such as administration of cyclophosphamide or gemcitabine-containing chemotherapy might improve the efficacy of peptide vaccines[79,80,81]. Gemcitabine was also revealed in a mouse model to eliminate splenic Gr-1$^+$/CD11b$^+$ myeloid suppressor cells that contributed to immunosuppression by inhibiting the function of CD8$^+$ T cells[81].

In contrast to the vaccination for patients with solid cancer or *de novo* acute leukemia, it may be better to reduce the peptide dosage for treatment of patients with MDS or CML so that malignant cells are damaged gradually, not rapidly, because hematopoiesis in these patients is mainly sustained by malignant hematopoietic stem or progenitor cell–derived mature cells, and therefore, rapid and strong destruction of these malignant cell–sustained hematopoiesis before recovery of normal hematopoiesis may induce pancytopenia in peripheral blood, as described before in this article (Fig. 2). In fact, vaccination of a chronic myelomonocytic leukemia (CMML) patient, a hematological stem cell desease, with a very low dose of the WT1 peptide could reduce abnormal immature hematopoietic cells without leukocytopenia[82] .

Possible strategies to improve the WT1 peptide vaccine from immunological aspect are summarized in Table 3.

**TABLE 3**
**Immunological Aspect-Based Strategies to Improve WT1 Peptide Vaccine**

| Strategies | Expected Effects |
|---|---|
| Use of stronger adjuvant | More activation of DCs at vaccine injection sites |
| Combined use with WT1-derived helper peptide | Activation of WT1-specific CD4$^+$ T cells to further activate WT1-specific CTLs and make "memory" CTLs |
| Use of multiple epitope peptides | Activation of different kinds of CTLs |
| Combined use with chemotherapy | Suppression of Treg and/or myeloid suppressor cells |
| Reduction of WT1 peptide dosage for MDS or CML | Gradual decrease of malignant cells to make enough time for recovery of normal hematopoiesis (hematopoiesis is mainly sustained by malignant cells in patients with the diseases.*) |

* See text for the details.

DC: dendritic cell, CTL: cytotoxic T lymphocyte, Treg: regulatory T-cell, MDS: myelodysplastic syndromes, CML: chronic myelogenous leukemia.

Translational research is composed of basic research and the subsequent clinical trials. The latter is based on the scientific data obtained from the former. In other words, the data obtained in the clinical trials have a scientific basis. Therefore, it is expected that we will be able to obtain valuable scientific information about immune responses against TAAs in tumor-bearing patients by analyzing the samples (blood, bone marrow, tumor, or vaccinated site) obtained from the patients treated with the WT1 peptide cancer vaccine. This information will be useful not only for understanding the immune responses against TAAs in tumor-bearing patients, but also for further improvement of the cancer vaccine.

Chemotherapy drugs directly attack cancer cells, while cancer vaccines do not. The latter activate the immune system to attack cancer cells. Therefore, we may need new "response evaluation criteria" specific for cancer vaccine therapy[83]. It is probable that some cancer patients treated with cancer vaccines will be able to survive long term without complete disappearance of the tumors and with good quality of life (QOL)[58,65], or that the tumor growth will be stabilized or even regress after a temporary increase in size because immunotherapy is generally not as quick acting as chemotherapy[83,84]. Therefore, when we evaluate the vaccination-induced clinical responses with the Response Evaluation Criteria in Solid Tumors (RECIST) system, which is a gold standard for evaluating the response of solid tumors in the field of cancer chemotherapy, it might be recommended that stable disease (SD) is more highly regarded in cancer immunotherapy than cancer chemotherapy, particularly when the SD periods persist for a long while, and that stabilization of the disease after temporary progression of disease, which is categorized as progressive disease (PD) in RECIST, is also evaluated favorably, like "stabilization after temporal PD", based on the maximal size at the time point of the cessation of tumor growth. Although we should not excessively expect efficacy of cancer vaccine, we should not underestimate the efficacy.

Once we accumulate evidences that show the ability of the WT1 peptide vaccine to suppress tumor growth in patients with rather high tumor burden, we should start clinical trials in "adjuvant settings" in which diseases are minimal after operation and/or chemotherapy. Adjuvant settings would be the most suitable settings for cancer vaccines to show their ability. Recurrence of the diseases might be significantly reduced by the vaccination.

## ACKNOWLEDGMENTS

This study was supported in part by a Grant-in-Aid from the Ministry of Education, Science, Sports and Culture and the Ministry of Health, Labor, and Welfare, Japan. We thank Ms. T. Umeda and Ms. Y. Watatani for their help in enrollment of patients for clinical trials and arrangement of the patients' samples and data. We also thank Ms. K. Hamada for preparation of this manuscript.
